# Effects of monovalent cations on folding kinetics of G-quadruplexes

**DOI:** 10.1042/BSR20170771

**Published:** 2017-07-12

**Authors:** Jing You, Hui Li, Xi-Ming Lu, Wei Li, Peng-Ye Wang, Shuo-Xing Dou, Xu-Guang Xi

**Affiliations:** 1 Beijing National Laboratory for Condensed Matter Physics and CAS Key Laboratory of Soft Matter Physics, Institute of Physics, Chinese Academy of Sciences, Beijing 100190, China; 2 School of Physical Sciences, University of Chinese Academy of Sciences, Beijing 100049, China; 3 College of Life Sciences, Northwest A&F University, Yangling 712100, China; 4 LBPA, IDA, ENS Cachan, CNRS, Université Paris-Saclay, Cachan F-94235, France

**Keywords:** G-quadruplex, monovalent cations, folding kinetics, stopped-flow

## Abstract

G-quadruplexes are special structures existing at the ends of human telomeres, the folding kinetics of which are essential for their functions, such as in the maintenance of genome stability and the protection of chromosome ends. In the present study, we investigated the folding kinetics of G-quadruplex in different monovalent cation environments and determined the detailed kinetic parameters for Na^+^- and K^+^-induced G-quadruplex folding, and for its structural transition from the basket-type Na^+^ form to the hybrid-type K^+^ form. More interestingly, although Li^+^ was often used in previous studies of G-quadruplex folding as a control ion supposed to have no effect, we have found that Li^+^ can actually influence the folding kinetics of both Na^+^- and K^+^-induced G-quadruplexes significantly and in different ways, by changing the folding fraction of Na^+^-induced G-quadruplexes and greatly increasing the folding rates of K^+^-induced G-quadruplexes. The present study may shed new light on the roles of monovalent cations in G-quadruplex folding and should be useful for further studies of the underlying folding mechanism.

## Introduction

Telomeres are structural regions composed of DNA and proteins at the ends of linear chromosomes in eukaryotic cells, playing significant roles in protecting the chromosome ends from deterioration, as well as protecting cells from aging [1]. Human telomeric DNA is consisted of tandem TTAGGG repeats, which can fold into compact G-quadruplex structures by Hoogsteen hydrogen bonds with four guanines forming a planar tetrad and three tetrads stacking further [2]. G-quadruplex structures have attracted great attention because of their physiological significance on telomeric regulation and maintenance [3], and their potentials as the target of anti-cancer drugs, such as platinum drugs [4].

There are several types of G-quadruplex structures formed by human telomeric repeats, such as parallel [5], antiparallel basket [6], antiparallel chair [7], (2+2) antiparallel [8], hybrid-1 [9,10], hybrid-2 [11], and 2-tetrad basket [12]. The structure of G-quadruplexes not only depends on the DNA sequence [13], but also is affected by the ionic environments. In the presence of Na^+^, G-quadruplex DNA (AGGG(TTAGGG)_3_) has been reported to fold predominantly in an antiparallel basket-type structure, with a 265-nm negative peak and a 290-nm positive peak as the characteristic peaks in its circular dichroism (CD) spectra [14]. In the presence of K^+^, G-quadruplex DNA mainly folds into hybrid structures, with a 235-nm negative peak and, 260- and 290-nm positive peaks [15]. It should be noted that, although great efforts have been made on the folding kinetics and pathways of G-quadruplexes, the detailed mechanism still remains elusive and controversial. For example, previous studies suggested that G-quadruplex folding undergoes a simple sequential pathway, involving up to two intermediate states [7,16–22], whereas more recent studies proposed the occurrence of more complex multi-pathway folding processes [23–28]. Owing to the polymorphism and complexity of G-quadruplex structures, further studies are needed on the folding kinetics of G-quadruplexes and on the effects of cations involved therein.

In addition, since Li^+^ is regarded as a monovalent cation having no effect on G-quadruplex folding, it has been commonly used in control experiments for studying G-quadruplexes [20,21,29–34]. However, until now there is actually no careful study about the influence of Li^+^ on G-quadruplex folding processes.

Here, by using stopped-flow fluorescence method, we investigated the folding kinetics of G-quadruplexes with human telomeric repeats in different monovalent cation environments. We not only carried out folding kinetic assays in both Na^+^ and K^+^ environments, but also studied the kinetics of conformational transition from Na^+^- to K^+^-induced G-quadruplex structures. Furthermore, we studied the effects of Li^+^ on the G-quadruplex folding kinetics in Na^+^ and K^+^ environments. Surprisingly, we found that Li^+^ influences the folding fraction of Na^+^-induced G-quadruplexes, and greatly increases the folding rates of K^+^-induced G-quadruplexes. Our results contribute to a deeper understanding of G-quadruplex folding kinetics in different monovalent cation environments and may be helpful for further *in vitro* and *in vivo* studies of G-quadruplex structures.

## Experimental

### DNA sequence and sample preparation

The G-quadruplex DNA sequence is A(GGGTTA)_3_GGG, which is a snippet of human telomere repeat motifs. In our stopped-flow assays, fluorescent label carboxyfluorescein (FAM) was attached to the 3′ end and Hex (hexachlorofluorescein) to the 5′ end of the G-quadruplex DNA sequence. G-quadruplex DNA, with or without fluorescent labels, was purchased from Invitrogen (Thermo Fisher Scientific Co., Shanghai, China), and diluted to 100 μM with an annealing buffer (10 mM Tris/HCl, pH 8.0) for using. All chemicals were reagent grade and were obtained from Sigma–Aldrich (Shanghai, China).

### Stopped-flow kinetics study

All the stopped-flow kinetic assays were carried out using a Bio-Logic SFM-400 mixer with a 1.5 × 1.5 mm quartz cell. The Bio-Logic MOS450/AF-CD optical system (Bio-Logic Science Instruments, France) was equipped with a 150-W mercury-xenon lamp.

In the G-quadruplex folding kinetic assay, when the single-stranded DNA (ssDNA) is folded into a G-quadruplex structure, the distance between FAM and Hex will decrease by several nanometers, enhancing the efficiency of fluorescence resonance energy transfer (FRET) between the two dye molecules. The fluorescent label FAM (donor) was excited at 492 nm (2-nm bandwidth) and its emission signal was monitored at 525 nm using a high pass filter with 20-nm bandwidth (D525/20, Chroma Technology Co., U.S.A.). All the measurements were carried out at room temperature (23°C).

To measure the folding kinetic of DNA, the reacting reagents (DNA and monovalent cations) were separately dissolved in Tris/HCl buffer (10 mM, pH 7.5) in two different syringes, and the reaction was initiated by the rapid mixing of them. There were 20 nM G-quadruplex DNA and varying concentrations of ions in the final reaction solution. All concentrations mentioned in the present study were after mixing. The kinetic folding curves represented averages of over five individual traces, then were analyzed using Bio-Kine (version 4.26, Bio-Logic) with double- or triple-exponential functions for those with two or three folding phases respectively. In the Na^+^ form to K^+^ form transition assay, the G-quadruplex DNA was diluted in 100 mM NaCl solution and annealed overnight (heated to 92°C for 2 min and cooled slowly to the room temperature), and then reacted with K^+^ of different concentrations. In the Li^+^-related assays, Li^+^ and the other cation (Na^+^ or K^+^) were premixed adequately, then mixed with the G-quadruplex DNA to initiate the folding process.

### Circular dichroism spectrum measurements

CD spectrum measurements were performed on the Bio-Logic MOS450/AF-CD optical system. The G-quadruplex DNA without fluorescent labels were diluted in reaction buffer (10 mM Tris/HCl buffer, pH 7.5) containing specific monovalent cations to 1 μM, and detected right after the manual mixing. For each measurement, 1.5 ml of solution with 1 μM G-quadruplex DNA was contained in a quartz cell of 1-cm optical path length. The measurements were recorded at each nanometer from 220 to 320 nm at room temperature. Each spectrum curve was the averages of ten measurements and smoothed with a Savitsky–Golay filter.

## Results

### G-quadruplex folding kinetics in Na^+^ environment

**Figure 1:**
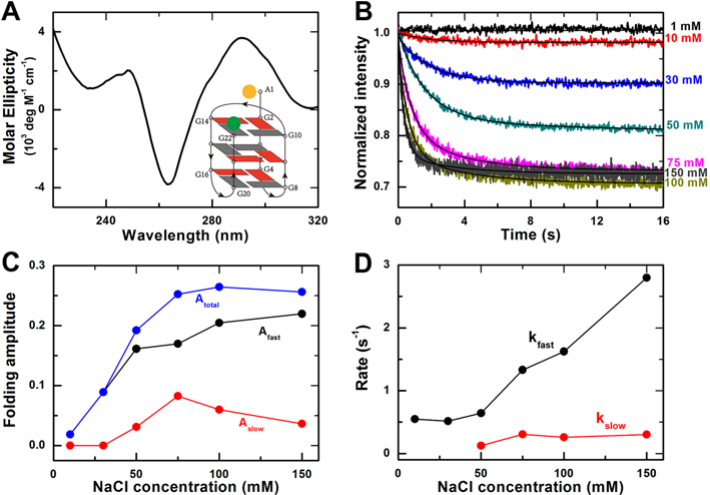
Effect of Na^+^ on G-quadruplex folding kinetics (**A**) CD spectra of G-quadruplexes in 100 mM NaCl buffer. Inset, G-quadruplex basket-type topology with fluorescent labels at the two ends of the DNA strand. (**B**) Time-courses of the fluorescence intensity of FAM after the unfolded G-quadruplex DNA were rapidly mixed with different concentrations of NaCl at room temperature (23°C). The solid lines are double-exponential fits of the data. (**C**) Folding amplitudes of the fast and the slow phases and the total amplitude obtained from the fittings in (B). (**D**) Folding rates of the fast and the slow phases from the fittings in (B).

In the presence of Na^+^, G-quadruplex DNA has been reported to fold predominantly in an antiparallel basket-type structure. To identify this structure, CD spectroscopy was carried out (Figure 1A). In accordance with previous studies, both a negative peak at 265 nm and a positive peak at 290 nm were observed, which are the characteristic peaks for the antiparallel basket-type structure [14]. The schematic view of this structure is shown in Figure 1 (A, inset).

The folding reactions were initiated by mixing rapidly the G-quadruplex ssDNA and NaCl of different concentrations (1, 10, 30, 50, 75, 100, and 150 mM). When the ssDNA molecules fold into G-quadruplex structures, the distance between the donor and acceptor would decrease, leading to increasing of the FRET efficiency. Accordingly, the fluorescence intensity of donor FAM would decrease and that of acceptor Hex would increase simultaneously (Supplementary Figure S1). In the following, we only monitored the fluorescence intensity of FAM for studying the G-quadruplex folding processes.

The G-quadruplex folding kinetic curves thus obtained were shown in Figure1 B. We found that these data curves can be well fitted by a double-exponential decay, suggesting that two phases are involved in the G-quadruplex folding process (see below). We used the decrease in fluorescence intensity or the folding amplitude to indicate the fraction of ssDNA that folded into the G-quadruplex structure.

As can be seen in Figure 1 C, the folding amplitude of the fast phase increases with increasing Na^+^ concentration and is almost saturated at 150 mM Na^+^, while that of the slow phase increases first with increasing Na^+^ concentration and then starts to decrease when the Na^+^ concentration is above 75 mM. The total amplitude increases with increasing Na^+^ concentration and is saturated at 100 mM Na^+^ (*A*_total_ = 0.265 ± 0.002), indicating that the fraction of DNA having folded into G-quadruplexes reaches the maximum at this Na^+^ concentration.

Similarly, the folding rate of the fast phase also increases, almost in a linear way, with increasing Na^+^ concentration (Figure 1D). At 100 mM Na^+^, which is close to the physiological concentration and used commonly in other studies, the rate constant is *k*_fast_ = 1.63 ± 0.02 s^−1^. In contrast, the folding rate of the slow phase remains almost constant and *k*_slow_ = 0.26 ± 0.01 s^−1^ at 100 mM Na^+^.

### G-quadruplex folding kinetics in K^+^ environment

**Figure 2:**
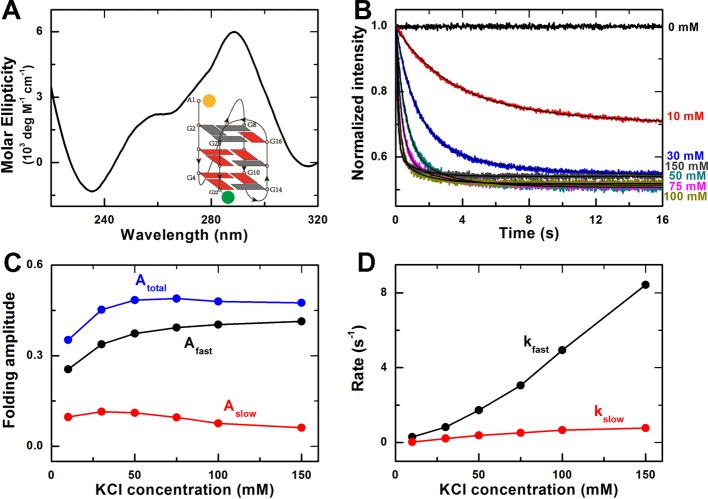
Effect of K^+^ on G-quadruplex folding kinetics (**A**) CD spectra of G-quadruplexes in 100 mM KCl buffer. Inset, G-quadruplex hybrid-1 type topology with fluorescent labels at the two ends of the DNA strand. (**B**) Time-courses of the fluorescence intensity of FAM after the unfolded G-quadruplex DNA were rapidly mixed with different concentrations of KCl at room temperature (23°C). The solid lines are double-exponential fits of the data. (**C**) Folding amplitudes of the fast and the slow phases, and the total amplitude. (**D**) Folding rates of the fast and the slow phases.

In the presence of K^+^, the negative peak at 235 nm and two positive peaks at 260 and 290 nm of the CD spectrum indicate the folding of the ssDNA into the hybrid G-quadruplex structures (Figure 2A), consistent with previous results [15]. There are two common hybrid G-quadruplex structures coexisting in K^+^ environment, hybrid-1 and hybrid-2, which could not be distinguished by CD spectra [7]. However, the hybrid-1 structure was identified as being thermodynamically favored [23,35], which may be the major structure in our experiment. The schematic view of the hybrid-1 structure is shown in Figure 2 (A, inset).

The folding kinetic assay was performed similarly as in the above Na^+^ experiments. The fast decrease in the fluorescence intensity of FAM shows that the ssDNA molecules folded more quickly in K^+^ environment (Figure 2B). The G-quadruplex folding kinetic curves can also be well fitted by a double-exponential decay. The overall trends of the data curves for the folding amplitude and rate are similar to that in the above Na^+^ case, but two main differences between the Na^+^ and K^+^ experiments may be noticed.

First, the total amplitude is saturated at 50 mM K^+^ with a greater value of *A*_total_ (0.482 ± 0.002) than that for Na^+^ (Figure 2C), suggesting that more ssDNA were folded into G-quadruplex structures in the presence of K^+^. This is consistent with previous single-molecule FRET experiments showing that almost all the DNA molecules were folded into G-quadruplex structures in K^+^ environment, whereas part of the DNA molecules was still unfolded in Na^+^ environment [29]. Note that the FRET efficiency is higher in the hybrid structure, as the distance between the donor and the acceptor is smaller than that in the antiparallel basket-type structure [13,29]. This might also contribute to the greater amplitudes in K^+^ environment.

Second, the folding in K^+^ environment was more rapid than that in Na^+^ environment. At 100 mM K^+^, the folding rates are *k*_fast_ = 4.94 ± 0.03 s^−1^ and *k*_slow_ = 0.66 ± 0.01 s^−1^ (Figure 2D). This suggests that K^+^ not only makes G-quadruplex structures more stable than Na^+^, but also has greater effect on the folding kinetics than Na^+^ at a given concentration.

Here, it should be mentioned that two phases were detected in both Na^+^- and K^+^-induced G-quadruplex folding kinetics. In recent studies that focused on the kinetic partitioning mechanism of G-quadruplex folding processes [24,26], it is indicated that unfolded ssDNA favors folding rapidly into multiple G-quadruplex structures, and then undergoes conformational reorganization to the final thermodynamically stable state. The two phases we observed may correspond to the above two processes and the kinetics parameters we obtained be the result of multiple reactions. This will be discussed later (see the first subsection of Discussion).

### Transition kinetics of G-quadruplex structures from Na^+^ form to K^+^ form

Previous studies revealed that the K^+^ ion could replace the Na^+^ ion inside a basket-type G-quadruplex structure and convert it to a hybrid-type form, whereas the opposite is not true, i.e. Na^+^ could not replace K^+^ inside a hybrid-type G-quadruplex structure and convert it to a basket-type form [6,7,15]. To further investigate the difference between Na^+^ and K^+^ on the G-quadruplex folding, we measured the transition kinetics of G-quadruplex structures from the Na^+^ form to the K^+^ form.

**Figure 3:**
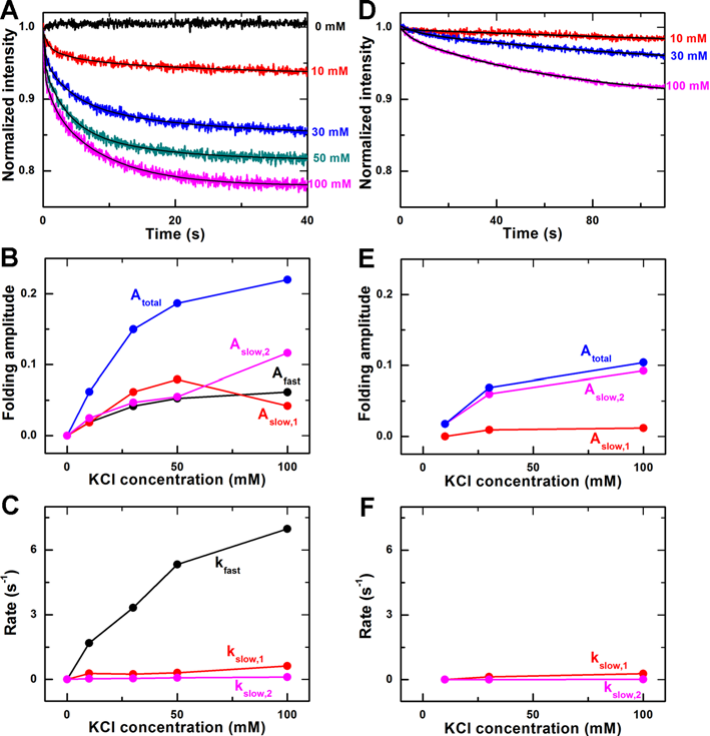
Transition kinetics of G-quadruplex structures from Na^+^ form to K^+^ form (**A**) Time-courses of the fluorescence intensity of FAM after the basket-type G-quadruplex, formed in the annealing buffer with 100 mM NaCl, were rapidly mixed with different concentrations of KCl at room temperature (23°C). The solid lines are triple-exponential fits of the data. (**B**) Folding amplitudes obtained from the fittings in (A). (**C**) Folding rates. (**D**) Time-courses measured in the same way as in (A) except for that 40% w/v polyethylene glycol-200 (PEG200) were added into the annealing buffer. The solid lines are double-exponential fits of the data. (**E**) Folding amplitudes in the case with PEG200. (**F**) Folding rates in the case with PEG200.

First, G-quadruplex ssDNA was annealed overnight in the annealing buffer containing 100 mM NaCl. Then, we monitored the donor’s fluorescence intensity right after the rapid mixing of the above DNA solution with KCl at different concentrations. The kinetic time-courses are shown in Figure 3A. Different from the above kinetic data in the Na^+^ or K^+^ experiments, the kinetic time courses for Na^+^−K^+^ transition are best fitted by a triple-exponential function, with one fast phase and two slow phases (designated as slow1 and slow2). As can be seen from the comparison in Supplementary Figure S2, the double-exponential fitting cannot describe the data well in the first second of the kinetic curve.

The folding amplitudes and rates obtained from triple-exponential fittings are given in Figure 3 B and C. As is expected, the total amplitude in the Na^+^–K^+^ transition assay (0.22 at 100 mM K^+^) is just coincident with the difference between the folding amplitude in 100 mM K^+^ environment (0.48, Figure 2C) and that in 100 mM Na^+^ environment (0.265, Figure 1C).

To figure out what the three phases represent, we performed control experiments by adding 40% w/v PEG200 into the 100 mM Na^+^ solution of G-quadruplex, to both provide the crowded environment and stabilize the folded G-quadruplex structures [5,36–39], prior to the Na^+^−K^+^ transition measurement. In this case, we found that the donor’s fluorescence intensity decreased slowly with time. Here, the fast phase disappeared and a double-exponential decay was sufficient to fit these intensity curves (Figure 3D). The corresponding folding amplitudes and rates thus obtained are given in Figure 3 E and F.

For the fast phase, as there are still unfolded ssDNA molecules in 100 mM Na^+^ environment (comparing the total folding amplitudes in Figures 1C and 2C) and almost all the ssDNA would fold in K^+^ environment [29], the fast phase we observed in the Na^+^−K^+^ transition experiment without PEG200 should correspond to a K^+^-induced folding of the previously unfolded ssDNA into G-quadruplex structure. When adding PEG200, the crowded environment would tend to compact the ssDNA molecules, promoting most unfolded ssDNA to fold into G-quadruplexes in the Na^+^ environment, as demonstrated by the increased characteristic peak values at 265 and 290 nm in CD spectra in previous studies [5,38]. The phenomenon was also observed in our experiment (Supplementary Figure S3). Therefore, the fast phase was then eliminated in the Na^+^−K^+^ transition experiment because, in the presence of PEG200, there was no longer unfolded ssDNA in the Na^+^ solution. Note that the folding rate of the fast phase in the Na^+^−K^+^ transition assay (6.97 s^−1^ at 100 mM K^+^, Figure 3C) is comparable with that in K^+^ environment (4.94 s^−1^ at 100 mM K^+^, Figure 2D), further indicating that the fast phase observed in the Na^+^−K^+^ transition experiment corresponds to K^+^-induced G-quadruplex folding.

The two slow phases of the decreasing fluorescence intensity may represent two phases in the conversion process of the G-quadruplex structures from the Na^+^ form to K^+^ form. The amplitudes and rates of the slow phases were also reduced by the crowded environment provided by PEG200 (Figure 3 E and F). It should be mentioned that PEG200 was reported to convert the K^+^-induced G-quadruplex structure from the hybrid-type to the parallel-type [37]. This conformational change results from the fact that PEG200 might specifically bind to the parallel-type K^+^-induced structure, rather than from the environment crowding [39], but this conformational change is a quite slow (hours-long) process [5]. Thus our measurements recorded within 100 s should mainly involve the conversion from the Na^+^-induced basket-type structure to the K^+^-induced hybrid-type structure in the crowded environment.

### G-quadruplex folding kinetics in Li^+^ and Na^+^ environment

Li^+^ cannot induce G-quadruplex folding and is usually used as a control monovalent cation for studying G-quadruplex folding in Na^+^ or K^+^ environments [20,21,30–34]. However, there is no information about the influence of Li^+^ on G-quadruplex folding kinetics, especially when combined with Na^+^ or K^+^. Therefore, we next carried out experiments focusing on Na^+^- or K^+^-induced G-quadruplex folding in the presence of Li^+^.

**Figure 4:**
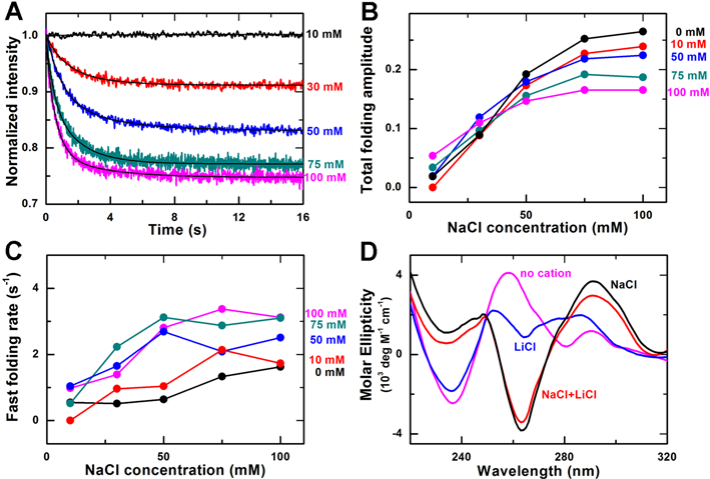
Effect of Li^+^ on Na^+^-induced G-quadruplex folding (**A**) Time-courses of the fluorescence intensity of FAM after the unfolded G-quadruplex DNA were rapidly mixed with different concentrations of NaCl and 10 mM LiCl at room temperature (23°C). The solid lines are double-exponential fits of the data. (**B**) Total folding amplitude versus NaCl concentration at different concentrations of LiCl. (**C**) Fast folding rate versus NaCl concentration at different concentrations of LiCl. (**D**) CD spectra of the G-quadruplex sequence in Tris/HCl buffer, 100 mM NaCl buffer, 100 mM LiCl buffer, and 100 mM NaCl buffer with100 mM LiCl.

First, we measured the G-quadruplex folding kinetics at different NaCl concentrations from 10 to 100 mM while the concentration of LiCl was fixed at 10 mM (Figure 4A). Then, we repeated the measurements by using higher LiCl concentrations (30, 50, 75, and 100 mM, Supplementary Figure S4). We found all these kinetic time-courses could be analyzed by a double-exponential function.

At a given concentration of Li^+^, the total folding amplitude of G-quadruplexes increases with increasing Na^+^ concentration (Figure 4B), just as in the case of Na^+^ only (Figure 1C), but interestingly, we noticed that the situation is not so simple at a given concentration of Na^+^. When the concentration of Na^+^ is low (10 mM), the folding amplitude increases with increasing Li^+^ concentration. However, when the concentration of Na^+^ is high (>30 mM), the folding amplitude becomes decreasing with increasing Li^+^ concentration. These results indicate that not only Li^+^ can influence the Na^+^-induced G-quadruplex folding, but also the underlying mechanism may be complicated, which are inconsistent with our previous knowledge that Li^+^ has no effect on G-quadruplex folding.

We think Li^+^ may influence the Na^+^-induced G-quadruplex folding in two ways. On the one hand, Li^+^ could have non-specific interactions with the phosphate groups of the DNA to cause DNA condensation [40], contributing to the Na^+^-induced G-quadruplex folding. On the other hand, Li^+^ could compete with Na^+^ by forming other structures different from G-quadruplexes, such as transient G-tetramers [41] or G-quartet with twisted G-quartet plates [34]. Since Na^+^-induced G-quadruplexes are not stable and may dynamically switch between the folding and unfolding states [29], Li^+^ may replace Na^+^ in the unfolded state, resulting in the formation of some other structures and thus reducing the fraction of Na^+^-induced G-quadruplex structure. In light of these observations, our observed phenomenon might be explained as follows.

When the concentration of Na^+^ is low (e.g. 10 mM), the fraction of Na^+^-induced G-quadruplexes is very low (Figure 1B), the condensation effect of Li^+^ on ssDNA is dominant, and the competitive effect of Li^+^ is not obvious since only few G-quadruplexes form. Thus, Li^+^ tends to enhance G-quadruplex folding and the folding amplitude increases with increasing Li^+^ concentration. On the contrary, when the concentration of Na^+^ is high, there are more formed G-quadruplexes. The effect of Li^+^-induced DNA condensation on G-quadruplex folding becomes unimportant (or redundant), whereas the competition effect of Li^+^ with Na^+^ becomes more obvious because more Na^+^-induced G-quadruplexes could act as the target of Li^+^. Thus as the Li^+^ concentration increases, the folding amplitude is decreased.

In addition to the folding amplitude, the folding rate of Na^+^-induced G-quadruplexes are also influenced by Li^+^. As shown in Figure 4C, the folding rate of the fast phase is generally enhanced by Li^+^ for a given Na^+^ environment. Enhancement as high as 4 folds may be observed.

It should be noted that another possible reason for the increased folding fraction of Na^+^-induced G-quadruplexes may be the structural change of G-quadruplexes caused by Li^+^, but our measurements of the CD spectra excluded this possibility. As shown in Figure 4D, the CD spectrum of the G-quadruplex DNA in 100 mM LiCl and 100 mM NaCl environment is very similar to that in only 100 mM NaCl environment. The positions of the 265 and 290 nm characteristic peaks are the same, and the CD values of the peaks are slightly reduced when adding Li^+^, demonstrating that Li^+^ does not change the Na^+^-induced G-quadruplex structure but only decrease its folding fraction. Interestingly, we also measured the CD spectra of the ssDNA in a buffer without any monovalent cations or in only 100 mM LiCl environment, and found that the two spectra were quite different. This indicates that Li^+^ alone may indeed induce the ssDNA to form some minor structures, i.e. transient G-tetramers.

### G-quadruplex folding kinetics in Li^+^ and K^+^ environment

**Figure 5:**
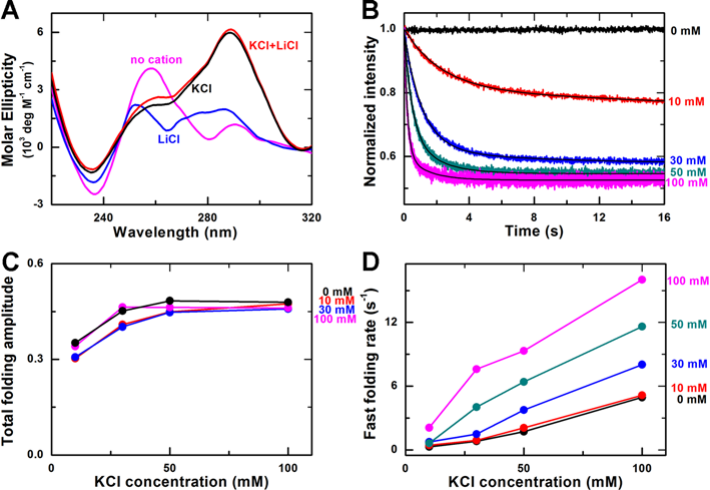
Effect of Li^+^ on K^+^-induced G-quadruplex folding (**A**) CD spectra of the G-quadruplex sequence in Tris/HCl buffer, 100 mM KCl buffer, 100 mM LiCl buffer, and 100 mM KCl buffer with 100 mM LiCl. (**B**) Time-courses of the fluorescence intensity of FAM after the unfolded G-quadruplex DNA were rapidly mixed with different concentrations of KCl and 10 mM LiCl at room temperature (23°C). The solid lines are double-exponential fits of the data. (**C**) Total folding amplitude versus KCl concentration at different concentrations of LiCl. (**D**) Fast folding rate versus KCl concentration at different concentrations of LiCl.

We next carried out experiments to study the influence of Li^+^ on kinetics of K^+^-induced G-quadruplex folding. Before the kinetic measurements, we first verified by CD spectra that Li^+^ does not change the K^+^-induced G-quadruplex structure (Figure 5A). For the kinetic measurements, we used similar method as that in the case of Li^+^ and Na^+^ environment above, and all the kinetic time-courses could also be analyzed by a double-exponential function (Figure 5B, Supplementary Figure S5).

Compared with the results in K^+^ environment (Figure 2), we noticed that the total folding amplitudes are almost independent of Li^+^ (Figure 5C), which is quite different from the observations in the above case of Li^+^ and Na^+^. This phenomenon can be explained as follows: (i) K^+^ is powerful to induce G-quadruplex folding even at 10 mM concentration, thus the condensation effect of Li^+^ on G-quadruplex folding is unimportant for the folding fraction; and (ii) K^+^-induced G-quadruplexes are of great stability compared with Na^+^-induced ones [41], which was also demonstrated in the above Na^+^−K^+^ transition results (Figure 3), so Li^+^ cannot compete with K^+^ by forming other structures different from G-quadruplexes.

Note that although Li^+^ has negligible effect on the folding fraction of K^+^-induced G-quadruplexes, Li^+^ enhances the folding rates of K^+^-induced G-quadruplexes. This should be attributed to the DNA condensation effect of Li^+^. In other words, the electropositive property of Li^+^ could neutralize the electrostatic repulsion of DNA and make it more flexible to fold [40]. The rate of the fast phase of K^+^-induced G-quadruplex folding is increased by nearly eight times in the presence of Li^+^ (Figure 5D), more notable than in the case of Na^+^-induced G-quadruplex folding. We believe one reason is that the competitive effect of Li^+^ takes effect in the latter case. Interestingly, by careful checking of the fast folding rates in different K^+^ and Li^+^ concentrations, we found that they are similar as long as the total ionic concentrations remain the same. As an example, the fast folding rate is 8.43 s^−1^ at 150 mM K^+^, 11.61 s^−1^ at 100 mM K^+^ and 50 mM Li^+^, and 9.33 s^−1^ at 50 mM K^+^ and 100 mM Li^+^. This indicates that the ionic strength plays a key role in the folding kinetics of G-quadruplexes.

## Discussion

Studies of the effects of monovalent cations on folding kinetics of G-quadruplexes with human telomeric repeats are essential for understanding the functions of telomeres. In the present study, we compared the effects of three monovalent cations (Li^+^, Na^+^, and K^+^) on G-quadruplex folding kinetics of the telomeric DNA sequence. The results are summarized as follows: (i) Both Na^+^ and K^+^ can induce G-quadruplex folding, but there is still unfolded ssDNA even in 150 mM Na^+^ environment whereas there is almost no unfolded ssDNA in only 50 mM K^+^ environment; (ii) K^+^ can replace Na^+^ inside Na^+^-induced G-quadruplexes, leading to structural transition from the basket-type form to the hybrid-type form, consistent with previous studies [15]; (iii) For Na^+^-induced G-quadruplex folding, Li^+^ can cooperate with Na^+^ to promote the folding at low Na^+^ concentrations, but compete with Na^+^ to reduce the folding fractions at high Na^+^ concentrations. The G-quadruplex folding rates are always enhanced by Li^+^; and (iv) For K^+^-induced G-quadruplex folding, Li^+^ has no obvious influence on the folding fraction but greatly promotes the folding rates.

### Reorganization in Na^+^- and K^+^-induced G-quadruplex folding

In both Na^+^- and K^+^-induced folding kinetics, a fast and a slow phase were detected. The fast phase may be attributed to the rapid folding of unfolded DNA into different intermediate conformational states, and the slow phase to the reorganization of these conformational states to reach the thermodynamic equilibrium, where there maybe exist, depending on the sequence of ssDNA and the buffer conditions, one or several G-quadruplex structures. For example, a 22-nt human telomeric sequence (Tel22) forms a single stable basket-type G-quadruplex in the presence of Na^+^ and a mixture of multiple G-quadruplex conformations in the presence of K^+^; however, a 26-nt human telomeric sequence (Tel26) does not form a well-defined unique G-quadruplex structure in the presence of Na^+^, but it forms a single stable hybrid-1 G-quadruplex in the presence of K^+^ [15].

Multiple intermediate states during G-quadruplex folding processes have been observed in many studies. In Na^+^ environment, it was observed that there are multiple G-quadruplex structures, such as antiparallel chair, (2+2) antiparallel and 2-tetra basket states, existing and interconverting even in high-concentration (up to 400 mM) NaCl solutions [29]. In another study, most intermediates were observed to switch into the antiparallel-basket type G-quadruplex structure, which is the most stable, in the final equilibrium state [6].

In K^+^ environment, more studies on the kinetic partitioning mechanism of G-quadruplex folding kinetics have demonstrated the existence of multiple G-quadruplex intermediates. Bessi et al. [23] have showed that the folding of Tel24 undergoes kinetic partitioning, and three types of structures are populated: hybrid-1, hybrid-2, and partially unfolded structures. The formation of the less stable hybrid-2 structure is kinetically favored. Refolding of the hybrid-2 structure involves extensive reorganization of the G-quadruplex to the more stable hybrid-1 structure via partially unfolded structures [23]. Marchand and Gabelica [25 ] have proposed that the unfolded G-quadruplex DNA may quickly misfold into antiparallel basket structure and then reorganize to hybrid structures. And Aznauryan et al*.* [24] have described a complex multi-pathway process of G-quadruplex folding that involves numerous folding/unfolding transitions and an extensive redistribution between several conformational states. Similarly, several molecular dynamics simulation studies also proposed that kinetically favored structures would form first and then transform into the more stable hybrid-1 structure [26–28]. All these studies suggested that the G-quadruplex ssDNA would first undergo the kinetically favored folding to form intermediate structures such as antiparallel, hybrid-2, or 2-tetrad G-quadruplex, and then these intermediate structures would reorganize to the final hybrid-1 structure in K^+^ environment.

Note that, Zhang and Balasubramanian [42] reported that the rapid folding of G-quadruplex was within 0.15 s, and Aznauryan et al*.* [24] identified the folding intermediates persisted 1–2 s. In our 100 mM K^+^ environment, the reaction time of the fast phase is 1/*k*_fast_ = 0.20 s and that of the slow phase is 1/*k*_slow_ = 1.51 s, which are consistent with the above reports. Moreover, with the increase in the K^+^ concentration, more hybrid-1 structures form directly from unfolded DNA, and the other intermediates become more unstable [24], so the folding amplitude would become lower and the folding rate higher for the slow phase, which are just what we observed (Figure 2C and D). As shown in Figure 1C, *A*_slow_ is equal to zero in 10 and 30 mM NaCl environments, i.e. there exists only one phase at low NaCl concentrations. We think the reason may be that, at low Na^+^ concentrations, the effect of Na^+^ on the G-quadruplex conformation is weak and thus there are no thermodynamically favored G-quadruplex structures other than the kinetically favored intermediate structures, therefore further conformational reorganization of the intermediate structures no longer occurs. In other words, these intermediate structures are essentially in equilibrium once they are formed.

It should be mentioned that, although G-hairpin [19] and G-triplex [18] were also supposed to be intermediate structures involved in the G-quadruplex folding process, these two structures could only exist within microseconds or milliseconds [21,42], beyond the temporal resolution of our measurements. Therefore, what we had mainly detected here should be the relatively long-lived intermediate states, the lifetimes of which were reported to be in seconds previously [29]. In addition, as our kinetic measurements were performed only within tens of seconds, the final state in our measurements might not be the most stable state of the folded G-quadruplex structures.

### Conformational transition from Na^+^- to K^+^-induced G-quadruplex structures occurs in two slow phases

In the conformational transition kinetic assay, G-quadruplex ssDNA annealed overnight in 100 mM NaCl were mixed rapidly with KCl at varying concentrations. We have shown that K^+^ could induce the unfolded fraction of ssDNA in Na^+^ solution to fold into G-quadruplex structures in a fast phase, and bring about structural transitions in two slow phases by replacing Na^+^. For the fast phase, Gray et al. [43] reported the jumping of CD signal at both 261 and 291 nm when measuring the kinetics of G-quadruplex structural switch induced by K^+^ within 5 s. We think the phenomenon is very likely caused by K^+^-induced folding of the unfolded ssDNA, as we observed here.

As we discussed above, in the Na^+^- or K^+^-induced folding process, the reorganization of different intermediate structures into the final structure(s) gives rise to the observed slow phase. However, the structural transition from Na^+^- to K^+^-induced G-quadruplexes occurs in two slow phases. This indicates that the G-quadruplex structural transition process is complicated. The reason may be that, both G-quadruplex structures (basket-type with Na^+^, hybrid-1 with K^+^) involved in the transition process are stable, whereas the intermediate structures in the Na^+^- or K^+^-induced folding processes are usually unstable.

Previously, Ambrus et al*.* proposed a structural switch pathway for the conformational transition from Na^+^- to K^+^-induced G-quadruplex structures, consisting of three steps [15]. In the first step, the K^+^ ions replace the Na^+^ ions inside the basket-type G-quadruplex structure, which causes no conformational change to the G-quadruplex structure and thus is undetectable in our kinetic measurements. In the next two steps, the presently unstable basket-type G-quadruplex structure partially unfolds, and then refolds into the hybrid-1 structure. We think the two slow phases that we observed in our transition assay probably correspond to these two steps.

### Influence of ionic strength on G-quadruplex folding kinetics

It is generally believed that Li^+^ has no effect on G-quadruplex folding and thus Li^+^ has been widely used as a control cation in studies of G-quadruplex folding. Our finding that Li^+^ may act as a cofactor of other cations and actually influence the folding kinetics of G-quadruplexes even at low concentrations is quite noteworthy. Li^+^ should not be used as a control ion in some cases, otherwise the results for G-quadruplex folding may be modified.

The ionic strength is important for the G-quadruplex folding through compacting DNA. The ions we used in the experiments are all monovalent ions, such as Na^+^, K^+^, Li^+^, and Cl^−^, so we can use the salt concentrations to evaluate the ionic strength approximately. Before addition of these salts, the ionic strength of the solution is 8.06 mM, which is from the 10 mM Tris/HCl buffer. After addition of the salts, the ionic strength can be considered as the total concentration of the salts (or Li^+^, Na^+^, and K^+^) by neglecting the low ionic strength from the Tris/HCl buffer.

To examine the influence of ionic strength on DNA condensation during the folding process, we carried out control experiments with dT_22_ (poly T sequence) in only Li^+^, Na^+^, or K^+^ environments, and with G-quadruplex ssDNA in only Li^+^ environment (Supplementary Figure S6A–D). In each of these cases, the fluorescence intensity remains the same during the 16-s measurement at a given ionic concentration; however, it decreases with increasing ionic concentration (Supplementary Figure S6E). This means that the ionic strength indeed induces the DNA condensation, which is, however, too fast to be detected in our measurements. In addition, the DNA condensation is positively correlated with the ionic concentration in each case.

From a comparison between the results for dT_22_ and those for the G-quadruplex ssDNA, it was found that Li^+^ has greater condensing effect on G-quadruplex ssDNA than dT_22_ (Supplementary Figure S6E). This is probably because the G-quadruplex ssDNA may quickly fold, aided by Li^+^, into minor structures, such as G-hairpin, leading to an increased FRET effect. The possibility of G-quadruplex formation is eliminated by the CD spectrum in the only Li^+^ environment given above (Figure 4D). Moreover, no change of fluorescence intensity that is characteristic of G-quadruplex folding was observed in the control experiment with only Li^+^ (Supplementary Figure S6D).

It should be pointed that the effect of ionic-concentration-dependent DNA condensation on the original fluorescence signals was obvious in all our kinetic measurements (see, for example, Supplementary Figure S6F), that is, the initial level of fluorescence intensity in each data curve always decreases with increasing ionic concentration, just as in the control experiment with only Li^+^ (Supplementary Figure S6D). However, this does not influence our final results because all the fluorescence intensity curves were normalized and, in addition, the kinetic processes we observed are only due to the folding of G-quadruplexes. Thus the variations of kinetic parameters we obtained for different ionic conditions should reflect solely their effects on G-quadruplex folding.

Finally, it should be noted that, in previous studies using the stopped-flow method, the G-quadruplex folding kinetics were investigated by measuring the CD signal [7] and the absorption signal [42]; whereas in the present study, we measured the fluorescence signal. Compared with the previous measurements, the fluorescence signal, which correlates with the FRET efficiency between the two dye molecules labeled on the G-quadruplex ssDNA, can report the change of distance between the two ends of the ssDNA more directly and sensitively during its structural transition to G-quadruplexes. From the measurements using the stopped-flow FRET method here, we were able to determine the G-quadruplex folding kinetic parameters in different ionic environments. The present experimental results may play an important role in future studies for further revealing the folding pathways and structural properties of G-quadruplexes.

## Supporting information

**Figure S1:** The FRET effect caused by G-quadruplex folding Fluorescence intensities of FAM (donor) at 525 nm and Hex (acceptor) at 595 nm verse time during the G-quadruplex folding process in 100 mM NaCl buffer at room temperature (23°C). The two measurements were performed separately (i.e., not simultaneously), because we had to change the optical filters. The solid lines are double-exponential fits of the data, yielding *A*_fast_ = 0.205 ± 0.003, *A*_slow_ = 0.059 ± 0.002, *k*_fast_ = 1.63 ± 0.02 s^−1^, and *k*_slow_ = 0.26 ± 0.01 s^−1^ for FAM; and *A*_fast_ = −0.193 ± 0.003, *A*_slow_ = −0.060 ± 0.001, *k*_fast_ = 1.52 ± 0.02 s^−1^, and *k*_slow_ = 0.22 ± 0.01 s^−1^ for Hex.

**Figure S2:** Double- or triple-exponential fittings of the transition kinetics of G-quadruplex structures from Na^+^ form to K^+^ form The black line is the time-course (only the initial 2 s was shown) of the fluorescence intensity of FAM after the basket-type G-quadruplex preformed in 100 mM NaCl was rapidly mixed with 100 mM KCl at room temperature (23°C). The red line represents a double-exponential fitting, yielding *A*_fast_ = 0.066 ± 0.001, *A*_slow_ = 0.128 ± 0.001, *k*_fast_ = 1.90 ± 0.04 s^−1^, and *k*_slow_ = 0.12 ± 0.01 s^−1^. The blue line represents a triple-exponential fitting, with *A*_fast_ = 0.061 ± 0.004, *A*_slow1_ = 0.042 ± 0.002, *A*_slow2_ = 0.117 ± 0.002, *k*_fast_ = 6.97 ± 0.08 s^−1^, *k*_slow1_ = 0.62 ± 0.04 s^−1^, and *k*_slow2_ = 0.11 ± 0.01 s^−1^.

**Figure S3:** Effect of PEG200 on Na+-induced G-quadruplex folding CD spectra of the G-quadruplex sequence in 100 mM NaCl buffer and 100 mM NaCl buffer with 40% PEG200 at room temperature (23°C).

**Figure S4:** Effect of Li^+^ on Na^+^-induced G-quadruplex folding Time-courses of the fluorescence intensity of FAM after the unfolded G-quadruplex DNA was rapidly mixed with different concentrations of NaCl and (A) 30 mM, (B) 50 mM, (C) 75 mM and (D) 100 mM LiCl at room temperature (23°C). The solid lines are double-exponential fits of the data, with some fitting parameters given in Figures 4B and 4C.

**Figure S5:** Effect of Li^+^ on K^+^-induced G-quadruplex folding Time-courses of the fluorescence intensity of FAM after the unfolded G-quadruplex DNA was rapidly mixed with different concentrations of KCl and (A) 30 mM, (B) 50 mM, and (C) 100 mM LiCl at room temperature (23°C). The solid lines are double-exponential fits of the data, with some fitting parameters given in Figures 5C and 5D.

**Figure S6:** Control experiments with dT_22_ and G-quadruplex ssDNA in different ionic environments, and one set of the original kinetic data curves Time-courses of the fluorescence intensity of FAM after the ssDNA rapidly mixed with different cations at room temperature (23°C). (A) dT_22_ with LiCl; (B) dT_22_ with NaCl; (C) dT_22_ with KCl; (D) G-quadruplex ssDNA with LiCl; (E) Fluorescence intensity versus the ionic concentration, where each data point was obtained by averaging all the data points in each of the kinetic curves shown above, and then normalized; (F) The original kinetic data curves corresponding to Figure 4A.

## Abbreviations

CD: circular dichroism
dT_22_: poly T sequence
FAM: carboxyfluorescein
FRET: fluorescence resonance energy transfer
Hex: hexachlorofluorescein
PEG200: polyethylene glycol-200
