# Supplementary material for: Effects of monovalent cations on folding kinetics of G-quadruplexes
Source: Biosci Rep. 2017 Jul 12;37(4):BSR20170771. doi: 10.1042/BSR20170771 (PMC5567087; doi:10.1042/BSR20170771)
Supplement: Supplementary file 1 [file bsr-37-bsr20170771-s1.pdf]

Supplementary materials

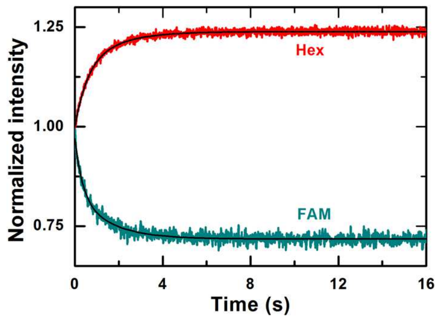

Figure S1 The FRET effect caused by G-quadruplex folding

Fluorescence intensities of FAM (donor) at 525 nm and Hex (acceptor) at 595 nm verse time during the G-quadruplex folding process in 100 mM NaCl buffer at room temperature (23 °C). The two measurements were performed separately (i.e., not simultaneously), because we had to change the optical filters. The solid lines are double-exponential fits of the data, yielding A_{fast} = 0.205 ± 0.003, A_{slow} = 0.059 ± 0.002, k_{fast} = 1.63 ± 0.02 s^{-1}, and k_{slow} = 0.26 ± 0.01 s^{-1} for FAM; and A_{fast} = –0.193 ± 0.003, A_{slow} = –0.060 ± 0.001, k_{fast} = 1.52 ± 0.02 s^{-1}, and k_{slow} = 0.22 ± 0.01 s^{-1} for Hex.

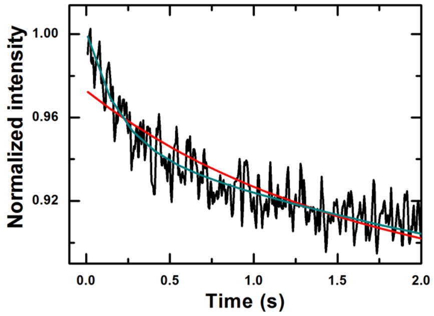

Figure S2 Double- or triple-exponential fittings of the transition kinetics of G-quadruplex structures from Na^+ form to K^+ form

The black line is the time-course (only the initial 2 s was shown) of the fluorescence intensity of FAM after the basket-type G-quadruplex preformed in 100 mM NaCl was rapidly mixed with 100 mM KCl at room temperature (23 °C). The red line represents a double-exponential fitting, yielding A_{fast} = 0.066 ± 0.001, A_{slow} = 0.128 ± 0.001, k_{fast} = 1.90 ± 0.04 s^{-1}, and k_{slow} = 0.12 ± 0.01 s^{-1}. The blue line represents a triple-exponential fitting, with A_{fast} = 0.061 ± 0.004, A_{slow1} = 0.042 ± 0.002, A_{slow2} = 0.117 ± 0.002, k_{fast} = 6.97 ± 0.08 s^{-1}, k_{slow1} = 0.62 ± 0.04 s^{-1}, and k_{slow2} = 0.11 ± 0.01 s^{-1}.

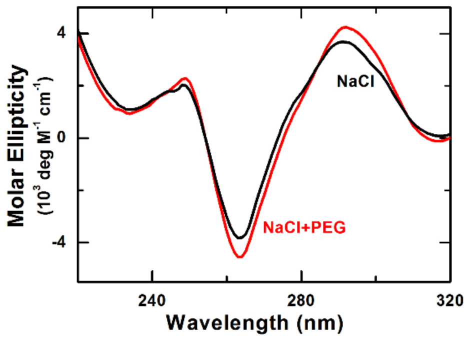

Figure S3 Effect of PEG200 on Na^{+}-induced G-quadruplex folding

CD spectra of the G-quadruplex sequence in 100 mM NaCl buffer and 100 mM NaCl buffer with 40% PEG200 at room temperature (23 °C).

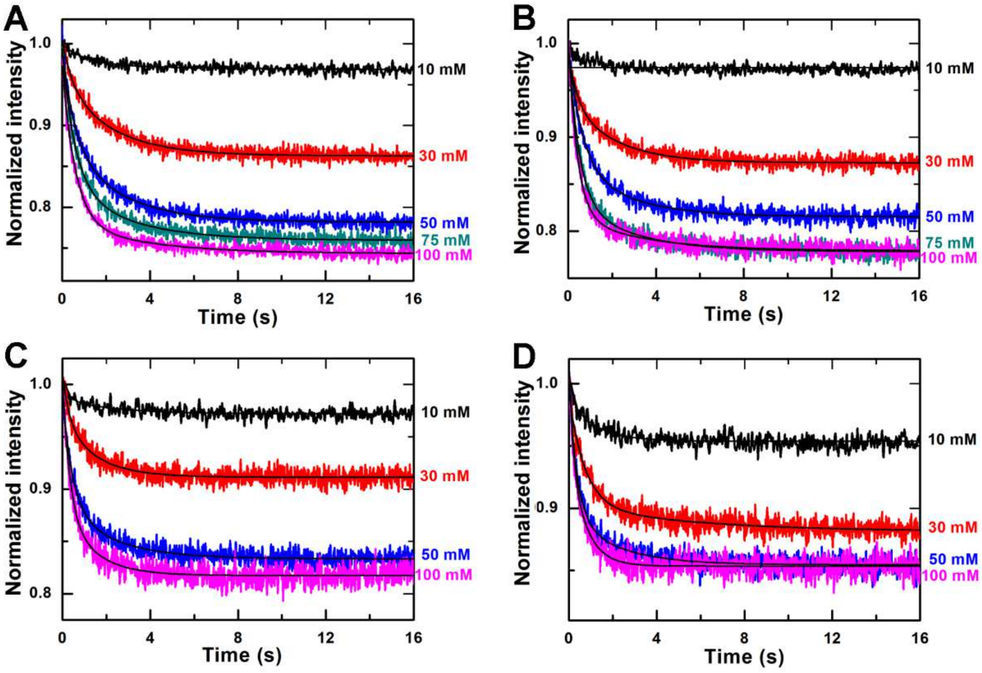

Figure S4 Effect of Li^+ on Na^+-induced G-quadruplex folding

Time-courses of the fluorescence intensity of FAM after the unfolded G-quadruplex DNA was rapidly mixed with different concentrations of NaCl and (A) 30 mM, (B) 50 mM, (C) 75 mM and (D) 100 mM LiCl at room temperature (23 °C). The solid lines are double-exponential fits of the data, with some fitting parameters given in Figures 4B and 4C.

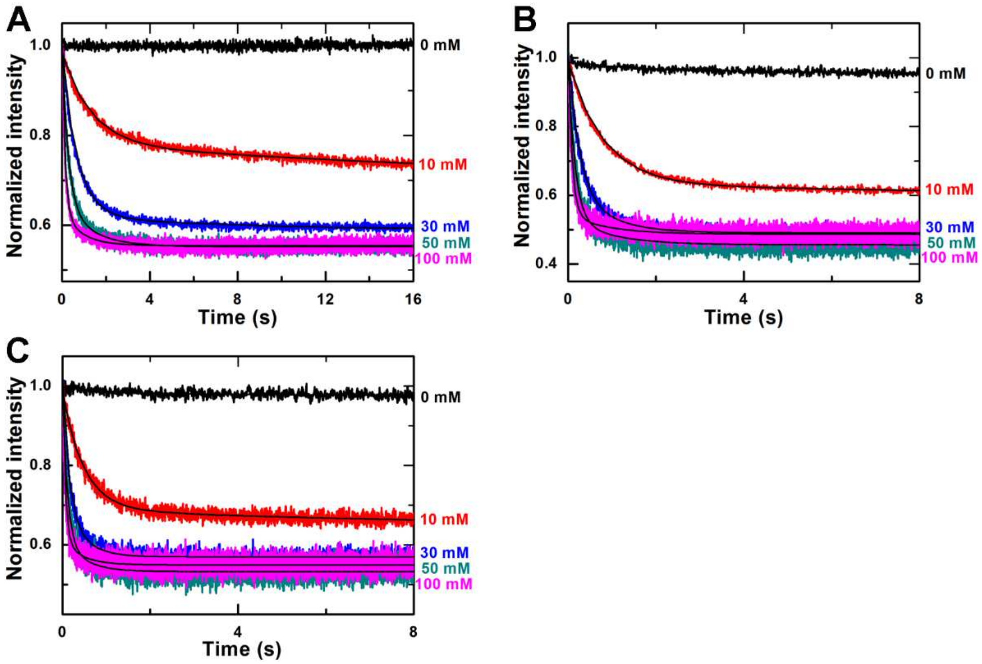

Figure S5 Effect of Li^+ on K^+-induced G-quadruplex folding

Time-courses of the fluorescence intensity of FAM after the unfolded G-quadruplex DNA was rapidly mixed with different concentrations of KCl and (A) 30 mM, (B) 50 mM, and (C) 100 mM LiCl at room temperature (23 °C). The solid lines are double-exponential fits of the data, with some fitting parameters given in Figures 5C and 5D.

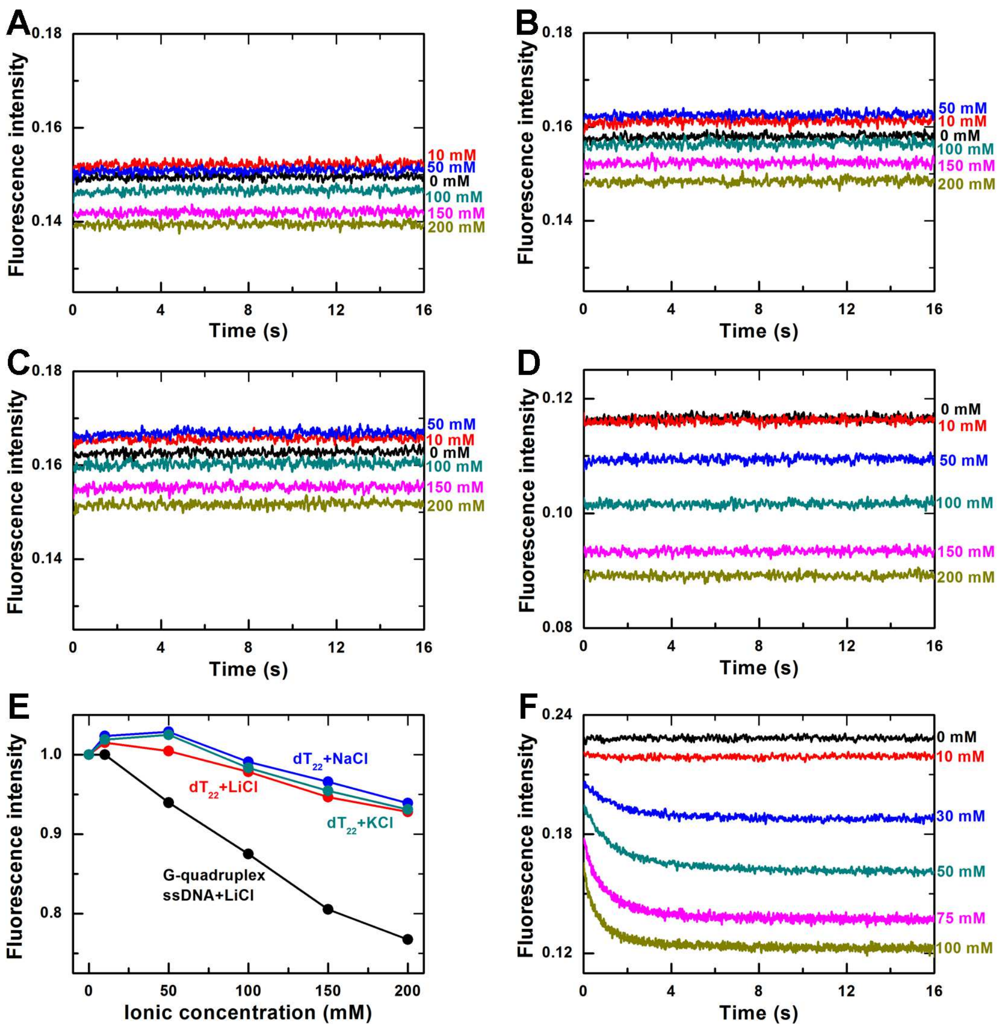

Figure S6 Control experiments with dT_{22} and G-quadruplex ssDNA in different ionic environments, and one set of the original kinetic data curves

Time-courses of the fluorescence intensity of FAM after the ssDNA rapidly mixed with different cations at room temperature (23 °C). (A) dT_{22} with LiCl; (B) dT_{22} with NaCl; (C) dT_{22} with KCl; (D) G-quadruplex ssDNA with LiCl; (E) Fluorescence intensity versus the ionic concentration, where each data point was obtained by averaging all the data points in each of the kinetic curves shown above, and then normalized; (F) The original kinetic data curves corresponding to Figure 4A.
